# Anti-FIRs (PUF60) auto-antibodies are detected in the sera of early-stage colon cancer patients

**DOI:** 10.18632/oncotarget.12696

**Published:** 2016-10-15

**Authors:** Sohei Kobayashi, Tyuji Hoshino, Takaki Hiwasa, Mamoru Satoh, Bahityar Rahmutulla, Sachio Tsuchida, Yuji Komukai, Tomoaki Tanaka, Hisahiro Matsubara, Hideaki Shimada, Fumio Nomura, Kazuyuki Matsushita

**Affiliations:** ^1^ Department of Molecular Diagnosis, Graduate School of Medicine, Chiba University, Chiba City, Chiba 260-8670, Japan; ^2^ Department of Physical Chemistry, Graduate School of Pharmaceutical Sciences, Chiba University, Chiba 260-8675, Japan; ^3^ Department of Biochemistry and Genetics, Graduate School of Medicine, Chiba University, Chiba 260-8670, Japan; ^4^ Divisions of Clinical Mass Spectrometry and Clinical Genetics, Chiba University Hospital, Chiba 260-8670, Japan; ^5^ Department of Molecular Oncology, Graduate School of Medicine, Chiba University, Chiba City, Chiba 260-8670, Japan; ^6^ Department of Academic Surgery, Graduate School of Medicine, Chiba University, Chiba 260-8670, Japan; ^7^ Department of Gastroenterological Surgery, Toho University Omori Medical Center, Tokyo 143-8541, Japan; ^8^ Department of Laboratory Medicine & Division of Clinical Genetics and Proteomics Chiba University Hospital, Chiba 260-8670, Japan

**Keywords:** auto-antibodies, cancer biomarker, colorectal cancer, far-upstream element-binding protein-interacting repressor (FIR) = poly(U)-binding-splicing factor (PUF60)

## Abstract

Anti-PUF60, poly(U)-binding-splicing factor, autoantibodies are reported to be detected in the sera of dermatomyositis and Sjogren's syndrome that occasionally associated with malignancies. PUF60 is identical with far-upstream element-binding protein-interacting repressor (FIR) that is a transcriptional repressor of *c-myc* gene. In colorectal cancers, a splicing variant of FIR that lacks exon2 (FIRΔexon2) is overexpressed as a dominant negative form of FIR. In this study, to reveal the presence and the significance of anti-FIRs (FIR/FIRΔexon2) antibodies in cancers were explored in the sera of colorectal and other cancer patients. Anti-FIRs antibodies were surely detected in the preoperative sera of 28 colorectal cancer patients (32.2% of positive rates), and the detection rate was significantly higher than that in healthy control sera (Mann–Whitney *U* test, *p* < 0.01). The level of anti-FIRs antibodies significantly decreased after the operation (*p* < 0.01). Anti-FIRs antibodies were detected in the sera of early-stage and/or recurrent colon cancer patients in which anti-p53 antibodies, CEA, and CA19-9 were not detected as well as in the sera of other cancer patients. Furthermore, the area under the curve of receiver operating characteristic for anti-FIRs antibodies was significantly larger (0.85) than that for anti-p53 antibodies or CA19-9. In conclusions, the combination of anti-FIRs antibodies with other clinically available tumor markers further improved the specificity and accuracy of cancer diagnosis.

## INTRODUCTION

A recent study reported that the detection of anti-PUF60, poly(U)-binding-splicing factor, auto-antibodies in dermatomyositis and Sjogren's syndrome, indicating it reflects the immune responses of the diseases [1]. On the contrary, the far-upstream element (FUSE)-binding protein-interacting repressor (FIR), splicing variant of PUF60 lacking exon5, have been reported to be overexpressed in various malignant tumors, such as colorectal cancers [2, 3], hepatocellular carcinomas [4, 5], T-cell acute lymphoblastic leukemia [6],and non-small cell lung cancer [7]. Therefore, it is natural that anti-FIR (PUF60) antibodies could be detected in the sera of cancer patients as well as in dermatomyositis and Sjogren's syndrome. So far, the significance of anti-FIR (PUF60) antibodies remains obscure in malignant complications of dermatomyositis or Sjogren's syndrome.

FIR is a c-Myc transcriptional repressor that is identical with PUF60. FUSE is a sequence required for the proper transcriptional regulation of the human *c-myc* [8]. c-Myc is critically activated in tumorigenesis in various tumors [9]. FUSE is located 1.5 kb upstream of the *c-myc* promoter P1 and recognized by FUSE-binding protein (FBP). FBP is a transcription factor that stimulates *c-myc* expression through FUSE [10–12]. Yeast two-hybrid analysis has demonstrated that FBP binds to FIR, and FIR represses *c-myc* transcription [13–16]. This study revealed that anti-FIRs antibodies were detected in gastrointestinal cancers. Therefore, anti-FIRs antibodies potentially reflect *c-myc* activation in auto-immune diseases and cancers.

## RESULTS

### Anti-FIR/FIRΔexon2 (FIRs) antibodies were detected in the sera of colorectal cancer patients

**Figure 1:**
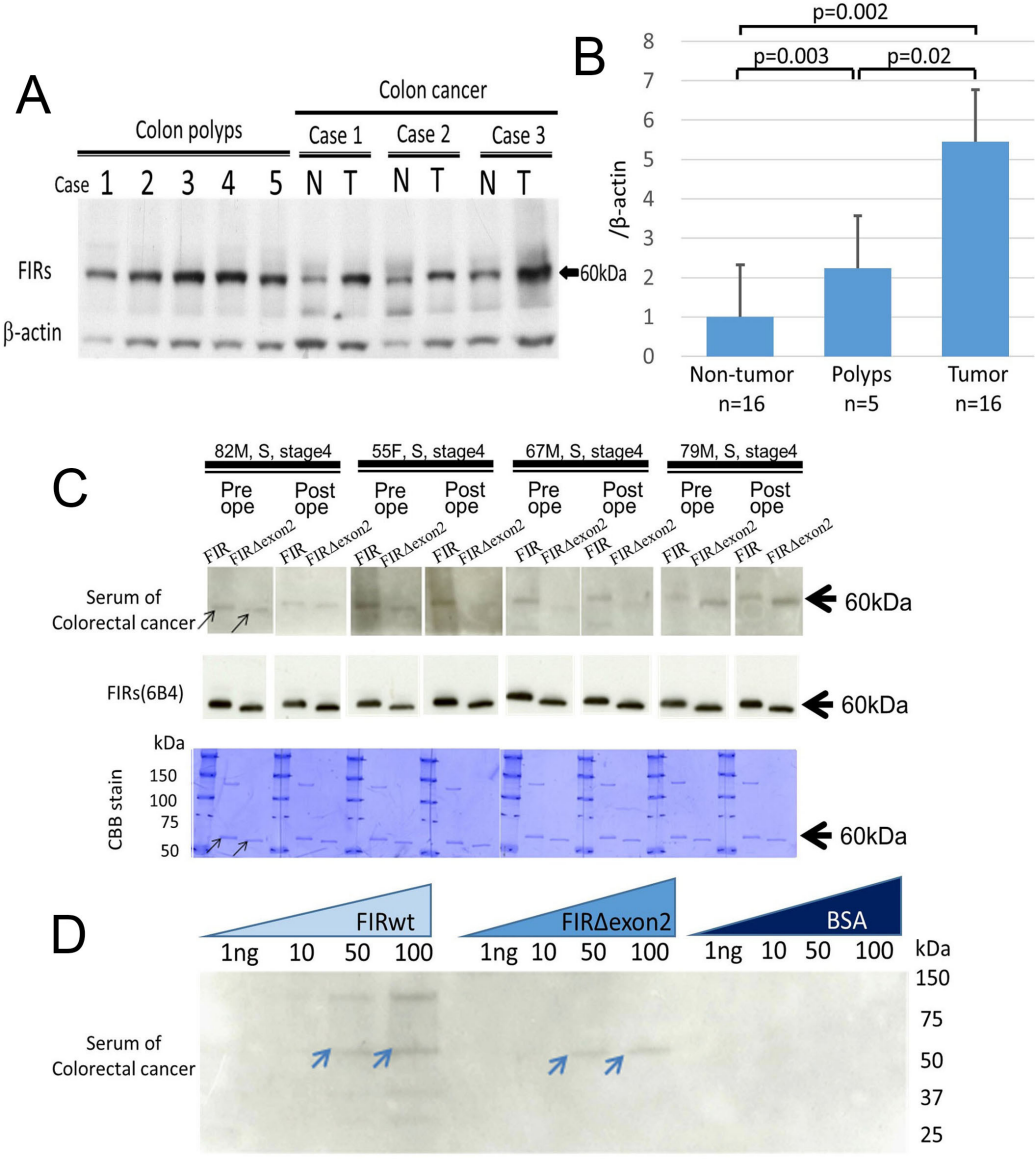
Auto-antibodies against FIR/FIRΔexon2 purified proteins were detected in the sera of colorectal cancer patients (**A**) Expression of FIRs proteins were examined by western blotting in tumor (T) and adjacent non-tumor (N) tissue samples from colon cancers and colon polyps' tissues. Representative cases were indicated. (**B**) Bands' intensities were quantified using Scion Image imaging analysis software (National Institutes of Health. USA) and the average band intensity of proteins normalized to the corresponding β-actin were shown. (**C**) FIR and FIRΔexon2 purified proteins were prepared as antigens to detect the auto-antibodies against FIR/FIRΔexon2 in colorectal cancer patients' sera by western blotting (upper panel). The anti-FIRs (6B4) antibody was used as a positive control (middle panel). Molecular weight of bands detected by patients' sera were exactly same size with those detected by anti-FIRs (6B4) antibodiy in western blot (middle panel) shown in CBB (Coomassie Brilliant Blue) staining (lower panel). (**D**) Auto-antibodies against FIR or FIRΔexon2 in colorectal cancer patients' sera were further confirmed in dose dependent manner by western blotting. FIRwt: FIR purified protein. BSA: Bovine serum albumin.

**Figure 2:**
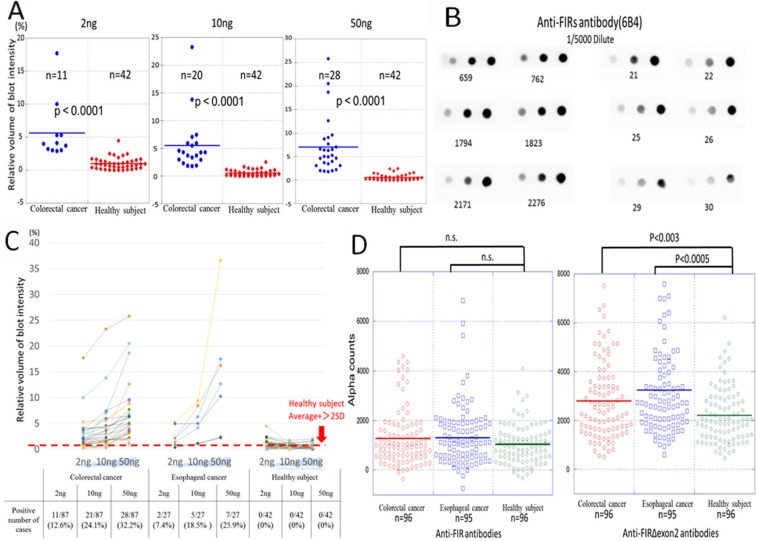
Detection of the anti-FIRs antibodies in the serum of colon cancer patients (**A**) The relative expression of anti-FIRs antibodies in the serum was detected against 2, 10, 50 ng of FIRΔexon2 purified proteins by dot blot analysis. The intensities of the dot signals were quantified by Lumi Vision Imager analysis software (Aisin Seiki Co., Ltd. Aichi, Japan). The relative intensity of the signals was compared between colorectal cancer patients and healthy subjects. (**B**) The dot blot membranes were stripped and reprobed with anti-FIRs (6B4) antibody for the confirmation of the dose of antigens. Dot blot intensities were standardized by positive control anti-FIRs (6B4) antibody. (**C**) Dose dependent curves of each patient were shown. (**D**) The levels of anti-FIRs antibodies in esophageal cancer (EC), colorectal cancer (CC) and were examined by AlphaLISA. Healthy subjects (HS) were as control. *P* values were calculated by Mann-Whitney *U* test.

FIR is a splice variant of PUF60 that lacks the exon 5 consists of 17 amino acids (Supplementary Figure S1A). In colorectal cancers, FIR is alternatively spliced lacking exon 2 (FIRΔexon2) that function as a dominant negative of authentic FIR [2] (Supplementary Figure S1A). FIRΔexon2/FIR mRNA is significantly elevated in colon cancer tissues [3]. The elevated FIRs expression has been reported to be overexpressed in various malignant tumors [2–7]. It has been reported that FIRs protein mainly located in the nucleus in colon cancers [3] and in hepatocellular carcinoma [5]. Interestingly, FIRs protein was overexpressed in adenomatous polyps and cancers of colon (Figure 1A and 1B and Supplementary Table S1, [3]). Further, a 60-kDa band (the molecular weight of FIR) and a 55-kDa band (the molecular weight of FIRΔexon2) were detected by western blot analysis with purified FIR/FIRΔexon2 as antigens in the colon cancer patients' sera as test-sets (Figure 1C, arrows). The bands were exactly overlapped with FIR/FIRΔexon2 proteins indicated by CBB staining (Figure 1D, arrows). Of note, the intensity of western blot was revealed to be in a dose-dependent manner (Figure 1D, arrows). These results strongly suggested that FIR/FIRΔexon2 antibodies were present in the sera of colorectal cancer patients. Subsequently, serum samples from 87 colorectal cancer patients, 27 esophageal cancer patients, and were examined by dot blot assay. Serum samples from 42 healthy volunteers were used as control. The representative pictures of dot blot assay indicated that FIR/FIRΔexon2 is present as an antigen in the sera of colorectal cancer patients (Supplementary Figure S2). The dot-blotted membranes were then stripped and incubated with purified anti-FIRs antibody (6B4) to confirm that handling inaccuracy was excluded (Figure 2A and 2B). The cutoff value of the positive blot intensity of cancer patients' serum was two times higher than that of the mean intensity of 42 healthy subjects (Figure 2C). The sensitivity of serum samples toward FIRs antigens was significantly higher in cancer patient groups than in controls. The sensitivity of anti-FIRΔexon2 antibodies was significantly higher than that of controls in colorectal (*p* < 0.0001) and esophageal cancer patients (*p* < 0.0027) (Figure 2D) detected by purified FIRΔexon2 proteins (Supplementary Figure S3). A positive predictive value of anti-FIRs antibodies in the sera of colorectal patients was 87% (Table 1).

**Table 1:** Comparison of biomarkers examined in this study of colon cancer patients

	Anti-FIRs antibodies	Anti-p53 antibodies	CEA	CA19-9
sensitivity(%)	78	30	78	62
specificity(%)	78	91	78	62
Positive Predictive Value(%)	87	87	88	77
Negative Predictive Value(%)	63	40	63	44

### Anti-FIRs antibodies were detected even in the early stages of colorectal and esophageal cancers

**Figure 3:**
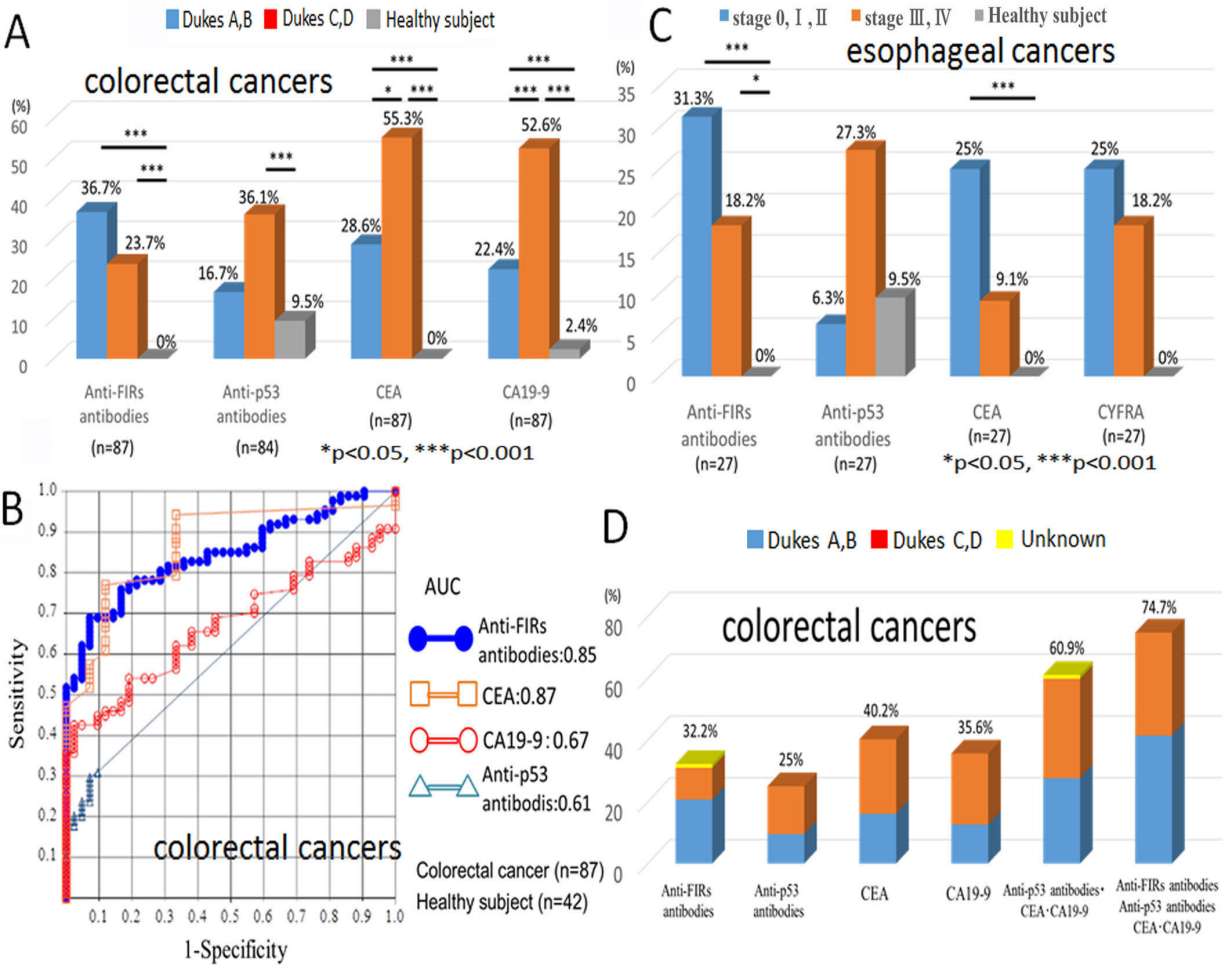
Diagnostic value of anti-FIRs antibodies in the sera of colorectal and esophageal cancer patients (**A**) The detection frequencies of anti-FIRs antibodies, His-tagged-FIRΔexon2 purified proteins as antigens, in the sera of early/advanced stages of colorectal cancer patients and healthy subjects were shown. (**B**) ROC (receiver operating characteristic) curve of colorectal cancer is indicated. The area under the curve (AUC) for anti-FIRs antibodies is 0.85, for anti-p53 antibody is 0.61, for CEA is 0.87 and for CA19-9 is 0.67. The best cutoff values for anti-FIRs antibodies, anti-p53 antibody, CEA and CA19-9 are 0.926%, 0.719 U/ml, 1.953 ng/ml and 12.8 U/ml, respectively. (**C**) The detection frequencies of anti-FIRs antibodies in the different stages of esophageal cancer patients and healthy subjects were also shown in graphical view. (**D**) The detection frequencies of anti-FIRs antibodies and three tumor markers in the different stages of colorectal cancer patients were shown in graphical view. The combination of three markers with anti-FIRs antibodies further increased the detection rate up to 74.7%.

Colorectal cancer patients were classified into early (Dukes stages A, B) and advanced (Dukes stages C, D) cancers. The percentage of anti-FIRs antibodies-positive cases were higher in early stage cancers (18/49, 36.7%) than in progressive cancers (9/38, 23.7%) (Figure 3A). Anti-p53 antibody, CEA, and CA19-9 were also examined in the serum. Unlike the anti-FIRs antibodies, these tumor markers were more frequently detected in advanced cancers (anti-p53 antibody: 13/36, 36.1%; CEA: 21/38, 55.3%; CA19-9: 20/38, 52.6%) than in early stage cancers (anti-p53 antibody: 8/48, 16.7%; CEA: 14/49, 28.6%; CA19-9: 11/49, 22.4%) (Figure 3A, 3B and Table 2). Based on the quantified dot blot data of 87 colorectal cancer patients and 42 healthy controls, an ROC curve of detected anti-FIRs antibodies and three clinically used tumor markers (anti-p53 antibody, CEA, and CA19-9) was examined (Figure 3C). There was no significant correlation among anti-FIRs antibodies with those three tumor markers, thereby anti-FIRs antibodies are independent markers for colorectal cancer (Supplementary Figure S4). The combined detection rate of anti-FIRs antibodies with anti-p53 antibodies, CEA and CA-19-9 indicated 74.7% (Figure 3D).

**Table 2:** Detection frequency of anti-FIRs antibodies and clinically used tumor markers in the sera of gastrointestinal carcinoma based on clinical stages

	Anti-FIRs antibodies	Anti-p53 antibodies	CEA	CA19-9	CYFRA
Colorectal cancer	28/87 (32.2%)	21/84 (25.0)	35/87 (40.2)	31/87 (35.6)	-
Dukes A,B	18/49 (36.7)	8/48 (16.7)	14/49 (28.6)	11/49 (22.4)	-
Dukes C,D	9/38 (23.7)	13/36 (36.1)	21/38 (55.3)	20/38 (52.6)	-
Esophageal cancer	7/27 (25.9)	4/27 (14.8)	5/27 (18.5)	-	6/27 (22.2)
stages 0. I. II	5/16 (31.3)	1/16 (6.3)	4/16 (25)	-	4/16 (25)
stages III. IV	2/11 (18.2)	3/11 (27.3)	1/11 (9.1)	-	2/11 (18.2)
Gastric cancer	0/20 (0)	-	-	-	-
stages 0. I. II	0/10 (0)	-	-	-	-
stages III. IV	0/10 (0)	-	-	-	-
Healthy subject	0/42 (0)	4/42 (9.5)	0/42 (0)	1/42 (2.4)	-

### The anti-FIRs antibodies in the sera decreased after colon cancer excision

**Figure 4:**
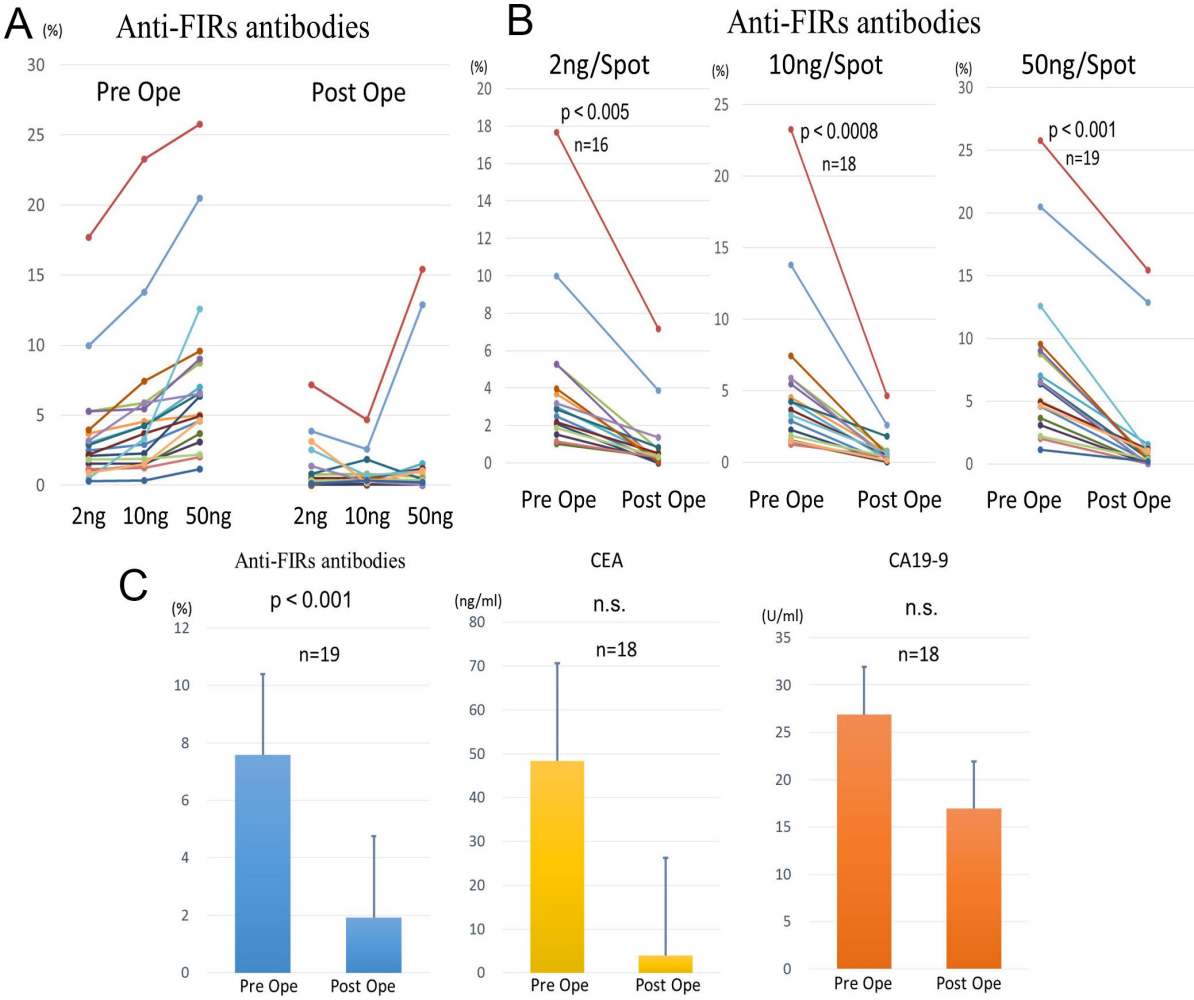
Evaluation of postoperative monitoring in colorectal cancer (**A**) Detection frequency of anti-FIRs antibodies in CRC sera before and after surgical operation. After surgical operation, anti-FIRs antibodies were detected in 19 cases out of 28 cases that were anti-FIR antibody positive before operation. (**B**) The level of anti-FIRs antibodies was significantly decreased after operation. (**C**) Comparison of the levels of anti-FIRs antibodies and clinically adopted tumor markers before and after surgical operation. The level of anti-FIRs antibodies was significantly decreased after operation.

**Figure 5:**
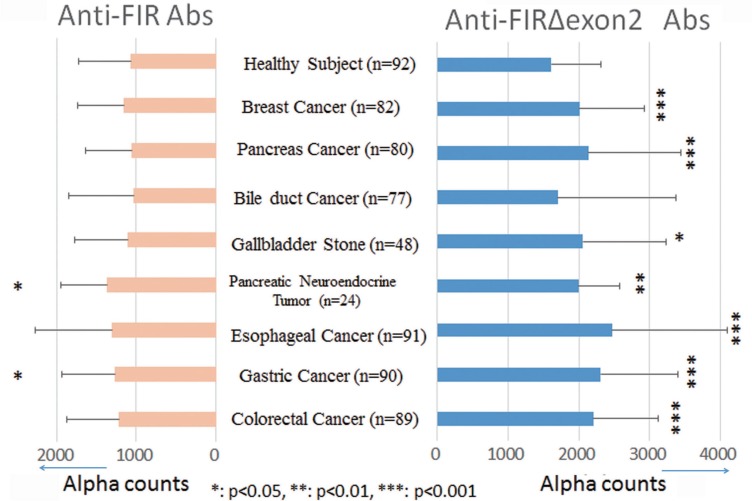
Anti-FIRΔexon2 or anti-FIR antibodies were detected in the sera of various cancer patients Purified proteins were prepared by Nus-tag FIR or -FIRΔexon2 as antigens. Alpha counts against FIR or FIRΔexon2 proteins were indicated in healthy subjects (*n* = 92), breast cancer (*n* = 82), pancreatic cancer (*n* = 80). bile duct cancer (*n* = 77), gall bladder stones (*n* = 48), pancreatic neuroendocrine tumor (*n* = 24), esophageal cancer (*n* = 91), gastric cancer (*n* = 90), and colorectal cancer (*n* = 89) patients.

We selected 19 out of 28 patients whose sera were determined as positive for anti-FIRs antibodies, according to the results of dot blot assay for 87 colorectal cancer cases. Serum samples from 19 patients were again collected after the absolutely complete surgical excision of the tumor. The expression level of anti-FIRs antibodies in the pre- and the post- operation was analyzed by a dot blot assay (Figure 4A). We compared the expression levels of pre- and postoperative anti-FIRs antibodies based on the antigen concentration. As a result, the expression levels of anti-FIRs antibodies significantly decreased after surgical treatment (antigen concentration 2 ng/spot, *p* < 0.005; 10 ng/spot, *p* < 0.0008; 50 ng/spot, *p* < 0.001) (Figure 4B). We also measured the levels of tumor markers CEA and CA19-9 in postoperative sera for reference. We observed a significant decrease in the level of anti-FIRs antibodies after surgical treatment, whereas the changes in the levels of CEA and CA19-9 were not significant (Figure 4C). As for screening sets, AlphaLISA assay was performed in the sera of other cancer patients to confirm the results. The levels of anti-FIR and -FIRΔexon2 antibodies were re-elevated along with cancer recurrences (Supplementary Figure S5). Anti-FIRΔexon2 were more sensitive than anti-FIR antibodies also in other cancers, such as pancreas, esophageal, gastric and colorectal cancers (Figure 5) detected by purified FIR or FIRΔexon2 proteins (Supplementary Figure S6). These results further suggested that anti-FIRs antibodies may also serve as a novel independent marker for the evaluation of postoperative monitoring and prognosis of cancers.

## DISCUSSION

In this study, purified full-length FIRΔexon2 protein (Supplementary Figure S1) was used as an antigen for western and dot blot analysis because it was more stable than full-length FIR protein (Supplementary Figure S3). The level of anti-FIRs antibodies in the sera of colon cancer patients was significantly more elevated than those of healthy controls (*p* < 0.01). Additionally, anti-FIRs antibodies were elevated in relatively early-stage colorectal cancer patients whose anti-p53 antibodies, CEA, and CA19-9 were below detection levels. Notably, FIRs were found in adenomatous polyps of colon (Figure 1A). Furthermore, the AUC of ROC for anti-FIRs antibodies was significantly larger than that for anti-p53 antibodies or CA19-9 (Figure 3B). Therefore, anti-FIRs antibodies are promising candidates for the diagnosis and postoperative monitoring of colon cancer patients (Supplementary Figure S1B). Altered FIR/FIRΔexon2 expression was detected as potential cancer-associated antigens in cancer tissues [3]. FIRΔexon2 activated *c-myc* mRNA expression whereas its levels were negatively correlated with interferon-gamma mRNA level which indicates local immune responses in cancer tissues [17]. Of note, anti-FIR/FIRΔexon2 antibodies were detected in the sera of early-stage cancer patients (Figure 3A, 3C). Together, anti-FIR/FIRΔexon2 antibodies could be helpful for detecting or discerning relatively early cancers especially in high risk population. Clinically, auto-antibodies have been applied for the diagnosis of colorectal cancer, breast cancer, and HCC [18, 19]. Anti-FIRs antibodies have different clinical profiles from anti-p53 antibodies (Figure 3C). The detection rates of colorectal cancers between anti-FIRs antibodies and anti-p53 antibodies were the same (Figure 3D); however, anti-FIRs antibodies were detected even in the early stages of cancer detection (Figure 3A). Some autoantibodies are reported to be detected in early cancer [21]. Therefore, the combination of anti-FIRs antibodies with anti-p53 antibodies, CEA, and CA19-9 is beneficial for colorectal cancer monitoring. The detection rates of colon cancer by anti-FIRs antibodies were almost the same rate as reported in meningioma cases by detecting the splicing variant of MGEA6/11 (41.7%) [20]. FIRΔexon2, a dominant negative form of FIR, competitively prevents FIR's ability to repress *c-myc* and contributes to *c-myc* transcriptional activation in several types of human cancers [5, 23]. Recently, anti-transcriptional intermediary factor 1-gamma (TIF1γ) /tripartite motif-containing protein 33 (TRIM33) antibodies have been detected in dermatomyositis patients with malignancies [24]. Since both FIR and TIF1γ/TRIM33 engage in Wnt-signaling pathway to suppress tumor progression [25–27], anti-FIRs antibodies could be helpful for the screening of dermatomyositis patients with malignancies as well as anti-TIF1γ/TRIM33 antibodies. Further examination is required in this field. In conclusion, anti-FIRs antibodies were detected in relatively early-stage colorectal cancers (Figures 1, 3, and Supplementary Figure S7). Therefore, the combination of anti-FIRs antibodies with other clinically available tumor markers increased the specificity and sensitivity for detecting colorectal cancers. Further, anti-FIRs antibodies could be applicable for detecting malignant tumors that sometimes associates with dermatomyositis or Sjogren's syndrome.

## MATERIALS AND METHODS

### Clinical samples

Human colorectal cancers and colon polyp tissues were obtained at the Department of General Surgery, Chiba University Hospital, Chiba, Japan. Written informed consent was obtained from all participants prior to this study. All excised cancer tissues were immediately placed in liquid nitrogen and stored at −80°C until analysis. Sera samples were obtained from: breast cancer (*n* = 82), pancreatic cancer (*n* = 80). bile duct cancer (*n* = 77), gall bladder stones (*n* = 48), pancreatic neuroendocrine tumor (*n* = 24), esophageal cancer (*n* = 91), gastric cancer (*n* = 89), colorectal cancer (*n* = 90) patients and healthy subjects (*n* = 92). These patients received surgical treatment in the Chiba University Hospital. Samples were collected during the pre- and postoperative periods. This study was conducted in accordance with “The Code of Ethics of the World Medical Association” (Declaration of Helsinki), and all study procedures were approved by the Ethics Committee of Chiba University.

### Preparation of purified full-length FIR or FIRΔexon2 proteins

A DNA code for FIR and/or FIRΔexon2 was cloned into the pET-50(b) expression vector (Novagen) (Supplementary Figure S3A). The pET-50(b) has the 6×His-conjugated Nus-tag and the HRV 3C cleavage site before its multiple cloning site. The Escherichia coli strain Rosetta (DE3) pLysS (Novagen), which was transformed with the pET-50(b) vector containing the FIR or FIRΔexon2 gene, was cultured in 4 L of lysogeny broth medium. The protein was expressed at 30°C overnight after induction with 0.2 mM isopropyl-β-D-thiogalactopyranoside (IPTG) at an OD600 value of 0.6. A cell pellet obtained via centrifugation of the cultured medium was resuspended in 40 mL buffer of 50 mM Tris-HCl (pH 8.0), 150 mM NaCl, 10 mM imidazole, and 1 mM phenylmethylsulfonyl fluoride (PMSF). The bacterial cell membrane was disrupted by sonication. After removing unnecessary disrupted fragments from lysate by using centrifugation, the expressed protein was obtained from the supernatant. The protein was first purified by a Ni-affinity column (HiTrap HP: GE healthcare) with a gradient rise of up to 500 mM in the imidazole concentration in elution buffer. The eluted protein fraction was dialyzed overnight against the buffer containing 50 mM Tris-HCl (pH 8.0) and 150 mM NaCl. The Nus-tag was then cleaved by HRV 3C protease for 24 h at 4°C. The cleaved protein was purified by a Co affinity column to remove the cleaved Nus-tag, the remaining uncleaved protein, and HRV 3C protease. The protein was purified for a third time by an anion exchange column with a gradient increase in the NaCl concentration in an elution buffer of 50 mM Tris-HCl (pH 8.0). The protein was finally purified by gel filtration (Hi-Load Superdex 200 pg, GE Healthcare) with a running buffer of 10 mM Tris-HCl (pH 8.0) and 150 mM NaCl. Purity of the sample was examined by electrophoresis with 10% polyacrylamide gel. The gel was stained by a fluorescent agent (Oriole, Bio-Rad) using a standard protein marker (Precision Plus, Bio-Rad). Molecular weights of FIR and FIRΔexon2 were 58,460 and 55,688, respectively (Supplementary Figure S3).

### Expression and purification of GST-fused FIR and FIRΔexon2 recombinant proteins

GST-fused FIR and FIRΔexon2 were expressed and purified for AlphaLISA measurement. As a reference, GST recombinant protein without FIR nor FIRΔexon2 was also obtained in the same manner. A DNA coding for GST-fused FIR and/or FIRΔexon2 was cloned into the pET-50(b) expression vector (Novagen) (Supplementary Figure S6). The *E. coli* strain, Rosetta (DE3) pLysS (Novagen), transformed with the pET-50(b) vector containing GST-fused FIR and/or FIRΔexon2 gene was cultured in 1L LB (Lysogeny Broth) medium. The protein was expressed at 30°C for overnight after induction at an OD600 value of 0.6 with 0.2 mM (IPTG). A cell pellet obtained with centrifugation of the cultured medium was resuspended in a 40mL buffer of 50 mM Tris-HCl at pH 8.0, 150 mM NaCl, 10mM imidazole, and 1mM (PMSF). Bacterial cell membrane was disrupted by sonication. After removing unnecessary disrupted fragments from lysate with centrifugation, the expressed protein was obtained from the supernatant. First, the protein was purified by a Ni-affinity column (HiTrap HP: GE healthcare) with a gradient rise of the concentration of imidazole in an elution buffer up to 500 mM. The eluted protein fraction was dialyzed overnight against the buffer without imidazole. Then, the Nus-tag was cleaved by HRV 3C protease for 24 hours at 4°C. The cleaved protein was purified by a Ni resin to remove the cleaved Nus-tag, the remaining uncleaved protein, and HRV 3C protease. The protein was finally purified by GST-affinity column with a step-wise increase of the concentration of reduced glutathione to 50 mM in an elution buffer of 50 mM Tris-HCl, 150 mM NaCl, and 1 mM ethylenediaminetetraacetic acid (EDTA) at pH 7.8. The purification was examined by an electrophoresis. Molecular weights of GST-fused FIR (793 a.a.) and FIRΔexon2 (764 a.a.) were 86,776 and 84,004, respectively.

### Western blot and dot blot analysis

To detect the anti-FIRs-antibodies in the sera samples, purified FIRΔexon2 protein was used as an antigen and diluted with 1× sodium dodecyl sulfate (SDS) sample buffer to the concentration of 50 ng/μL. The molecular size of anti-FIR/FIRΔexon2 (FIRs) autoantibodies or expression of FIRs proteins was confirmed by Western blot analysis as described previously [13]. Anti-FIRs antibodies were also detected by dot blot analysis and quantified using the Bio-Rad Immuno-Blot assay kit and the Bio-Dot microfiltration apparatus (Bio-Rad Laboratories, Hercules, California 94547 USA). This enzyme immunoassay optimizes the detection of a specific antigen by immobilizing the antigen on PVDF membranes (dot blot). All assay procedures were performed according to the manufacturer's instructions. In brief, the PVDF membrane was hydrophilized via incubation with 100% methanol for 15 min with continuous shaking, followed by incubation with 1×Tris-buffered saline (TBS)/0.1% Tween 20 for 15 min with continuous shaking. Purified FIRΔexon2 protein was diluted with 0.1% trifluoroacetic acid to the concentrations of 2, 10, and 50 ng/μL. These three concentrations of purified FIRΔexon2 protein were applied onto the hydrophilized PVDF membrane assembled in the Bio-Dot apparatus. The PVDF membrane was cut into appropriate sizes and blocked with 0.5% skimmed milk in 1×PBS/0.1% Tween 20 overnight at 4°C. Resized membranes were incubated with patient's serum (1:2000 dilution) for 1 h at room temperature, followed by three 10-min washes with 1×PBS/0.01% Tween 20. Membranes were then incubated with commercial secondary antibody (1:10000 diluted anti-human IgG antibody), followed by three 15-min washes with 1×PBS/0.01% Tween 20. The membranes were incubated in Western blot detection reagent (Pierce ECL Plus Western Blotting Substrate, 32132, Thermo Fisher Scientific, Waltham, MA). Stained bands were detected using a LPR-400EX chemiluminescence imager (Taitec, Tokyo, Japan) [28–31].

### AlphaLISA

The AlphaLISA method was used to evaluate the antibody levels in sera. The serum specimens used were obtained from 96 healthy subjects, 96 gastric cancer patients, 96 colon carcinoma patients, and 96 esophageal carcinoma patients. AlphaLISA was performed in 384-well microtiter plates (white opaque OptiPlate™, Perkin Elmer, Waltham, MA, USA) containing 2.5 μL of 1:100-diluted serum and 2.5 μL of GST-fusion antigen proteins (10 μg/mL) in AlphaLISA buffer (25 mM HEPES, pH 7.4, 0.1% casein, 0.5% Triton X-100, 1 mg/mL Dextran 500, and 0.05% Proclin 300). The reaction mixture was incubated at room temperature for 6–8 h, mixed with anti-human IgG-conjugated acceptor beads (2.5 μL at 40 μg/mL), and glutathione-conjugated donor beads (2.5 μL at 40 μg/mL), and then incubated for seven days at room temperature in the dark. The chemical emission was read on an EnSpire Alpha microplate reader (PerkinElmer) as described previously [32–34]. Specific reactions were calculated by subtracting Alpha values of GST control from the values of GST-fusion proteins.

### Statistical analysis

Fisher's exact (two-sided) probability test and the Mann–Whitney *U* test were used to determine the significance of the differences between the two groups. All statistical analyses were carried out using StatFlex software version 6.0 (Artech, Osaka, Japan). *p* < 0.05 was considered statistically significant.

### Serum tumor marker (anti-p53 antibody, CEA, and CA19-9 and CYFRA) measurement

Anti-p53 antibody was measured by ELISA using AP-960 (Kyowa Medx, Tokyo, Japan) with a cutoff value of 1.30 U/mL. CEA, CA19-9 and CYFRA were measured by CLEIA using a Lumipulse Presto II (Fujirebio, Tokyo, Japan), with cutoff values of 5.2 ng/mL, 36.8 U/mL and 2.1 ng/mL, respectively.

### Receiver operating characteristic (ROC) curves

The overall diagnostic efficiencies of anti-FIR antibodies, CEA, CA19-9, and anti-p53 antibodies were evaluated by comparing the ROC curves. The area under each ROC curve was calculated, and the statistical significance of the difference between ROC curves was assessed as described previously [35]. *p* < 0.05 was considered significant. ROC curves were generated, and the area under the curve (AUC) values were calculated using StatFlex software version 6.0.

## SUPPLEMENTARY MATERIALS


