# Supplementary material for: Anti-FIRs (PUF60) auto-antibodies are detected in the sera of early-stage colon cancer patients
Source: Oncotarget. 2016 Oct 15;7(50):82493–503. doi: 10.18632/oncotarget.12696 (PMC5347708; doi:10.18632/oncotarget.12696)
Supplement: Supplementary file 1 [file oncotarget-07-82493-s001.pdf]

Anti-FIRs (PUF60) auto-antibodies are detected in the sera of early-stage colon cancer patients

Supplementary Materials

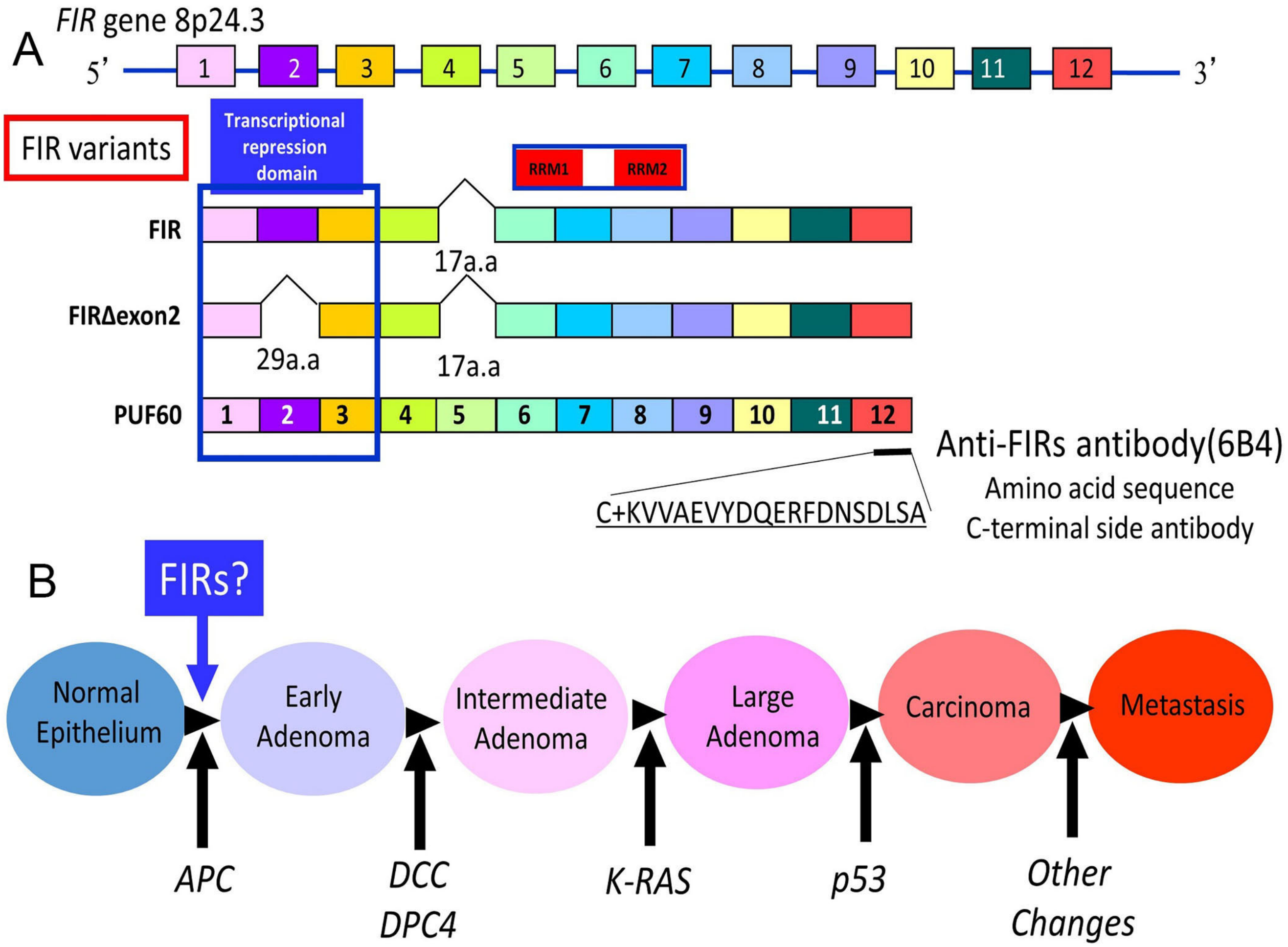

Supplementary Figure S1: (**A**) Schematic view of splice variants of the FIR gene. (B) The adenoma-carcinoma sequence. Progression of FIRs is accumulated in adenoma.

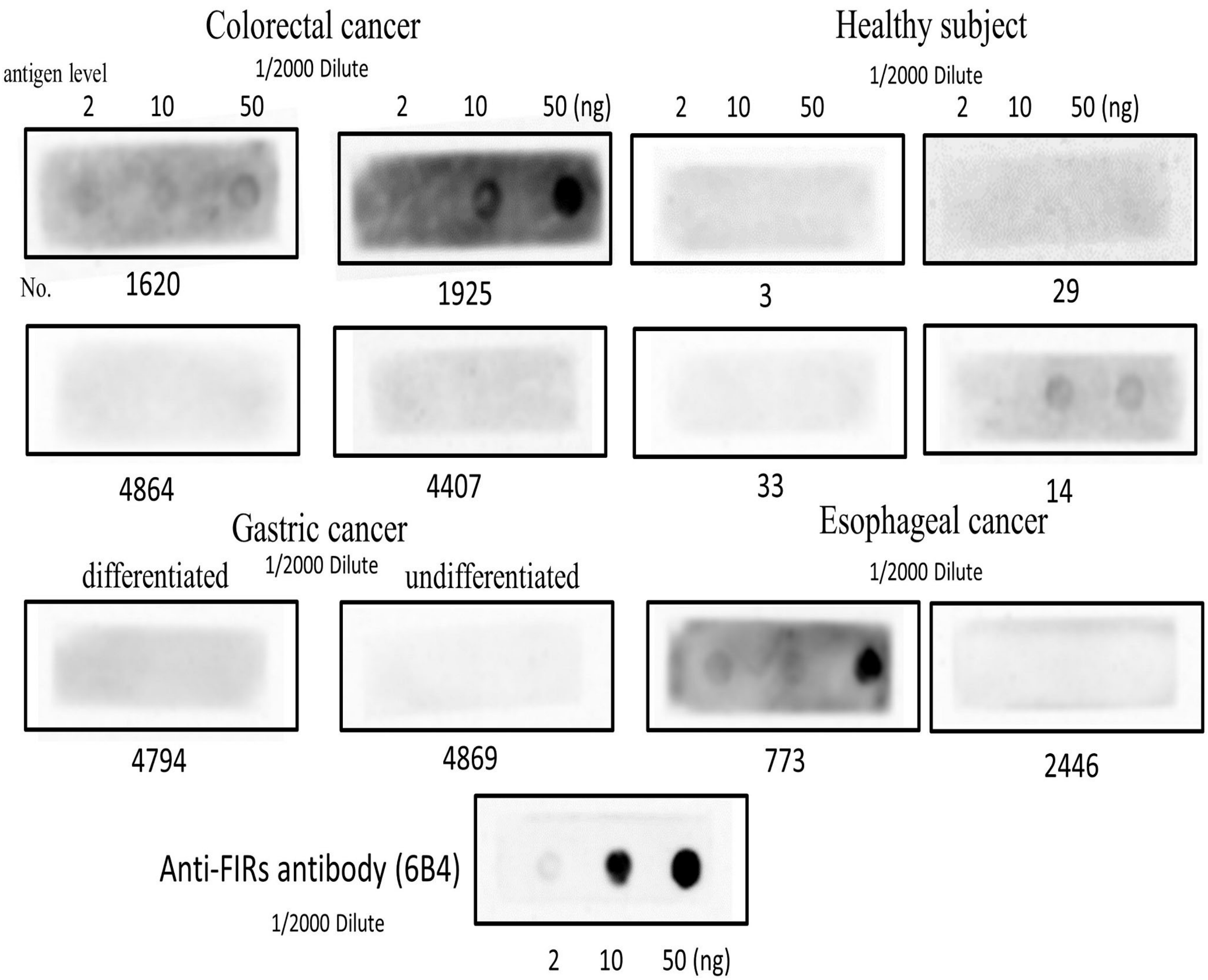

Supplementary Figure S2: Purified FIRΔexon2 protein was used as antigen to detect the possible presence of anti-FIR antibodies in the sera of colorectal cancer patients in a dose-dependent manner by dot blot assay.

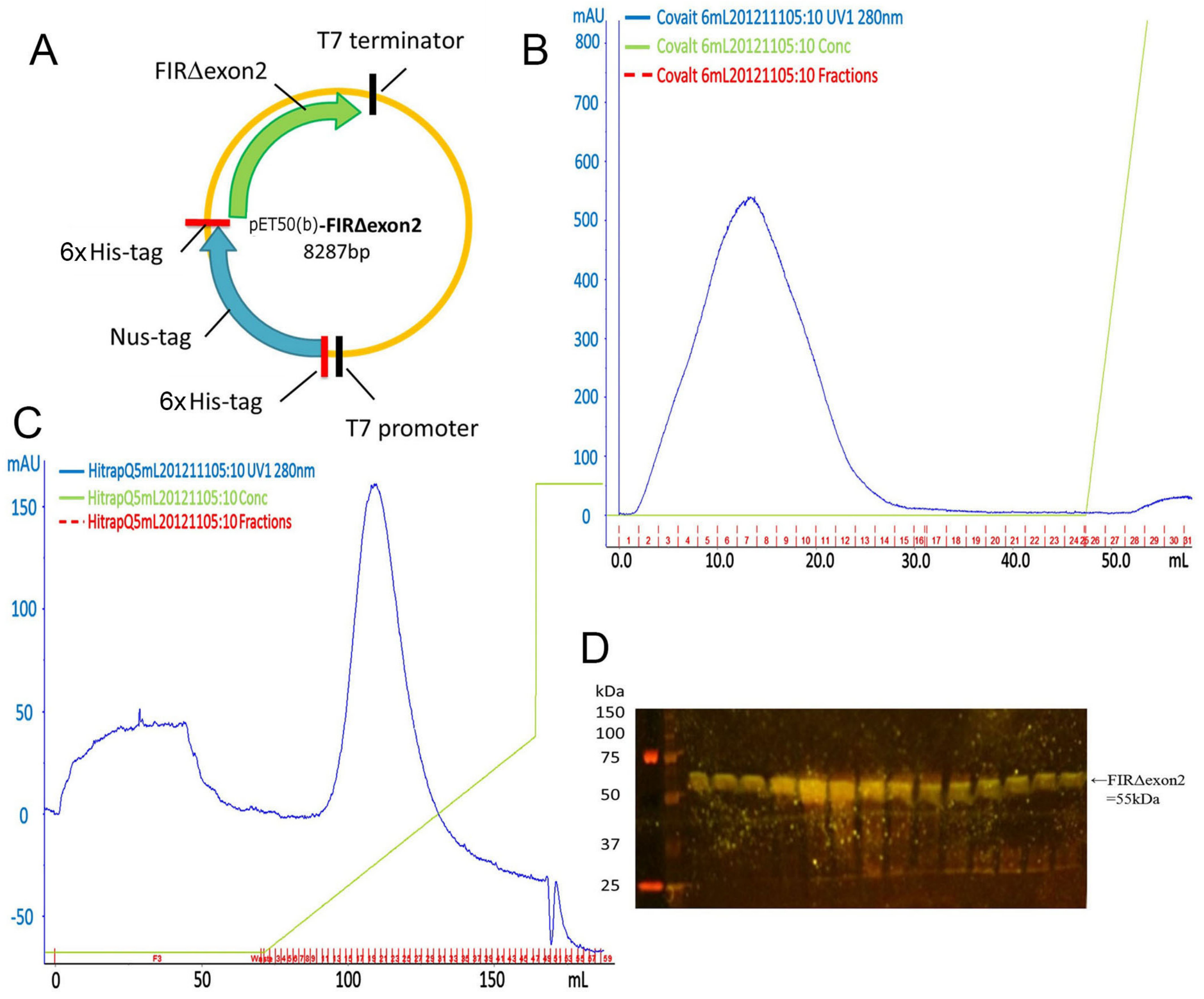

Supplementary Figure S3: (**A**) Expression vector pET50b (+) -FIRΔexon2. pET50b (+) -FIRΔexon2 expression vector was constructed by inserting the FIRΔexon2 gene into the pET-50b (+) DNA (Novagen) vector. (**B**) Co-affinity chromatography of purified FIRΔexon2 protein at the second phase. Imidazole concentrations are shown in green, and absorbance of 280 nm ultraviolet rays is shown in blue (0% = 10 mM, 100% = 500 mM). (**C**) The NaCl concentration of anion column chromatography of purified FIRΔexon2 protein is shown in green, and absorbance of 280 nm ultraviolet rays is shown in blue (0% = 50 mM, 100% = 1000 mM). (**D**) A 55.7-kDa band, which is the molecular weight of FIRΔexon2, was confirmed by gel electrophoresis after the His-Nus tag excision.

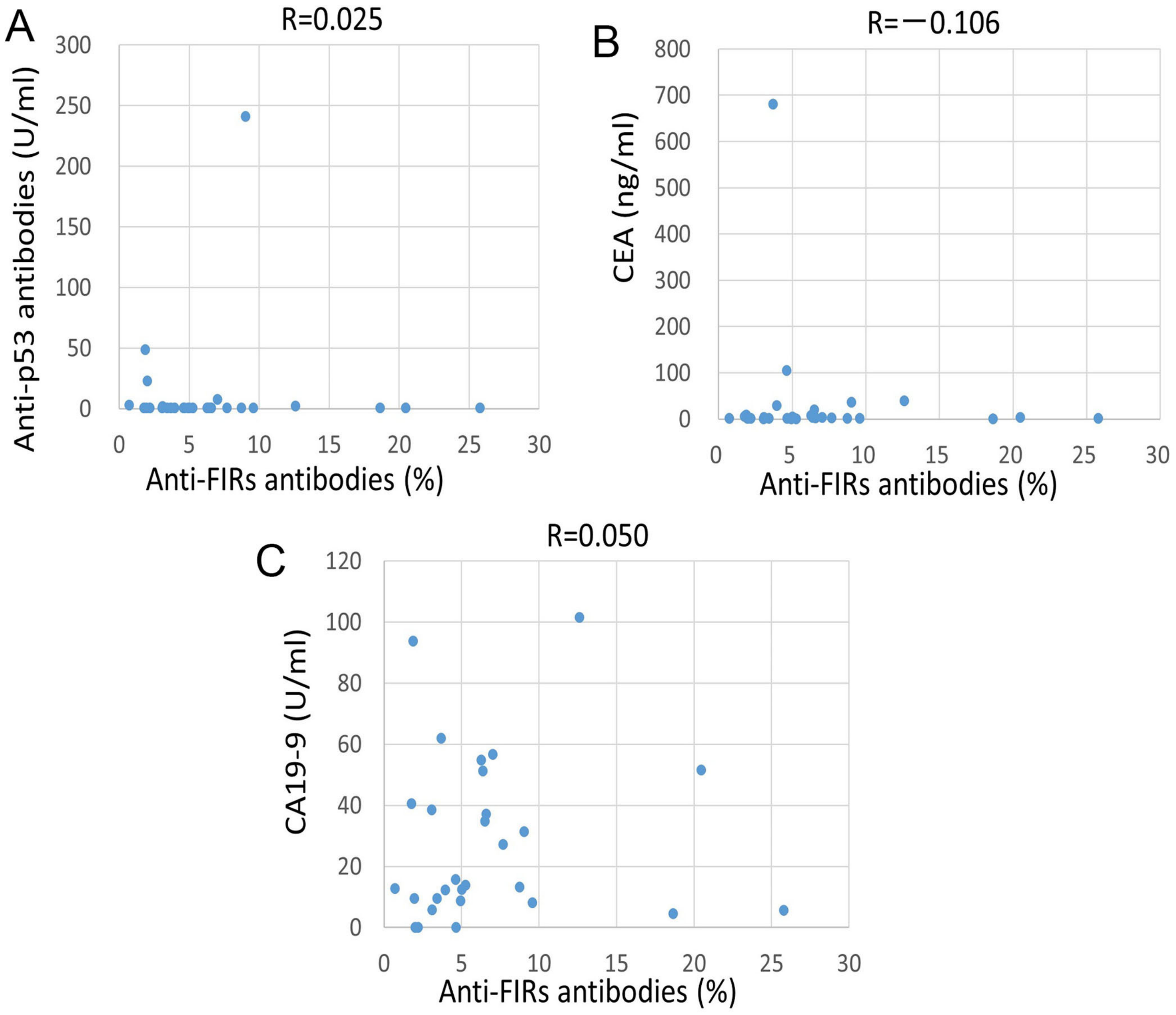

Supplementary Figure S4: Correlation between anti-FIR antibodies and well-known other tumor markers. Correlation between anti-FIR antibodies and three well-known tumor markers was analyzed by using statistical graphics. The level of anti-FIR antibodies detected in the sera of colorectal cancer patients was expressed as a percentage volume in the X-axis (horizontal axis). (**A**) The correlation between anti-FIR antibodies and anti-p53 antibodies. The level of anti-p53 antibodies measured in the sera of colorectal cancer patients was labeled in the Y-axis (vertical axis). (**B**) The correlation between anti-FIR antibodies and tumor marker CEA. The level of CEA measured in the sera of colorectal cancer patients was labeled in the Y-axis. (**C**) The correlation between anti-FIR antibodies and tumor marker CA19-9. The level of CA19-9 antibody measured in the sera of colorectal cancer patients was labeled in the Y-axis.

A

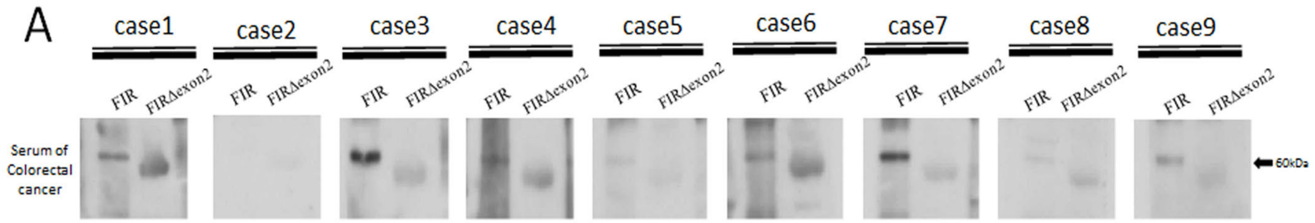

B

Clinical data	Age: 70s	sex: Female	Ascending colon cancer	Dukes D
	Pre ope Day 0	Post ope Day 35	Recurrence Day 450	Unit
Anti-FIR antibodies	5489	3124	6260	Alpha counts
Anti-FIRΔexon2 antibodies	5687	5000	6402	Alpha counts
CEA	265.0	245.0	153.5	ng/ml
CA19-9	77.8	78.2	156.4	U/ml
Anti-p53 antibodies	<0.20	-	-	U/ml

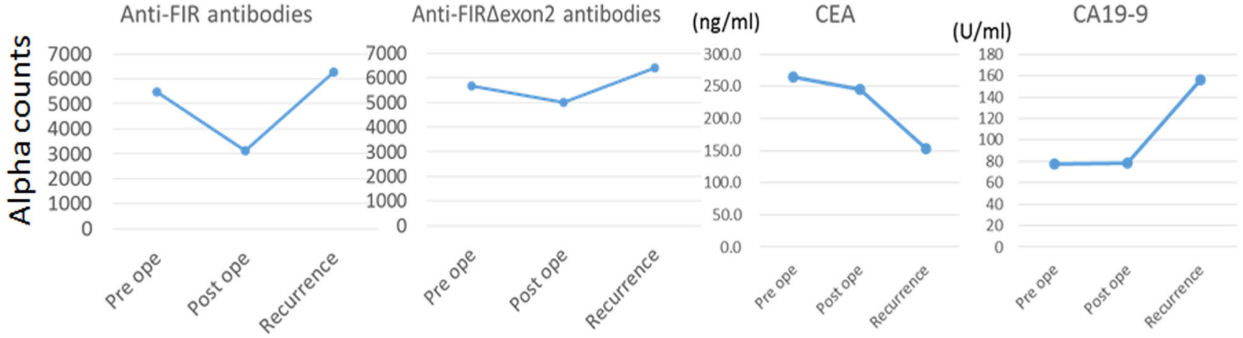

Supplementary Figure S5: (**A**) Anti-FIR or anti-FIRΔexon2 antibodies against Nus-tag FIR or -FIRΔexon2 as antigens were detected in nine relapsed cases of colon cancer patients after the operation. (**B**) The changes of anti-FIR or anti-FIRΔexon2 antibodies, CEA, and CA19-9 in case 9 before and after operation with relapse of cancers.

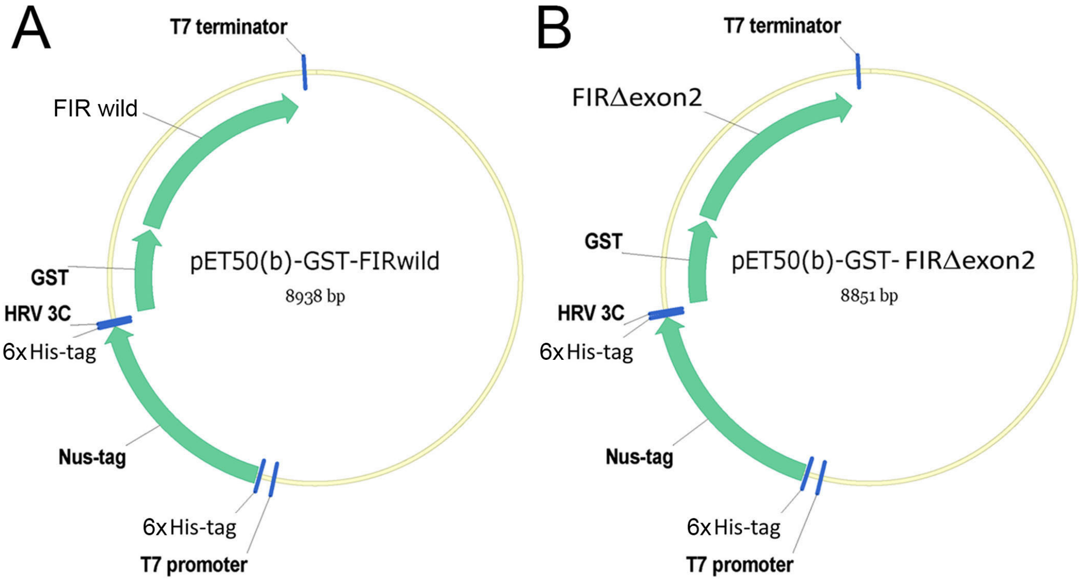

Supplementary Figure S6: Vector map for GST-fused FIR and FIRΔexon2 in the pET-50(b) expression vector. Both of the GST-fused FIR (**A**) and FIRΔexon2 (**B**) were expressed in the Nus-tag conjugated form and the Nus-tag was removed by the cleavage with HRV-3C enzyme in the purification process.

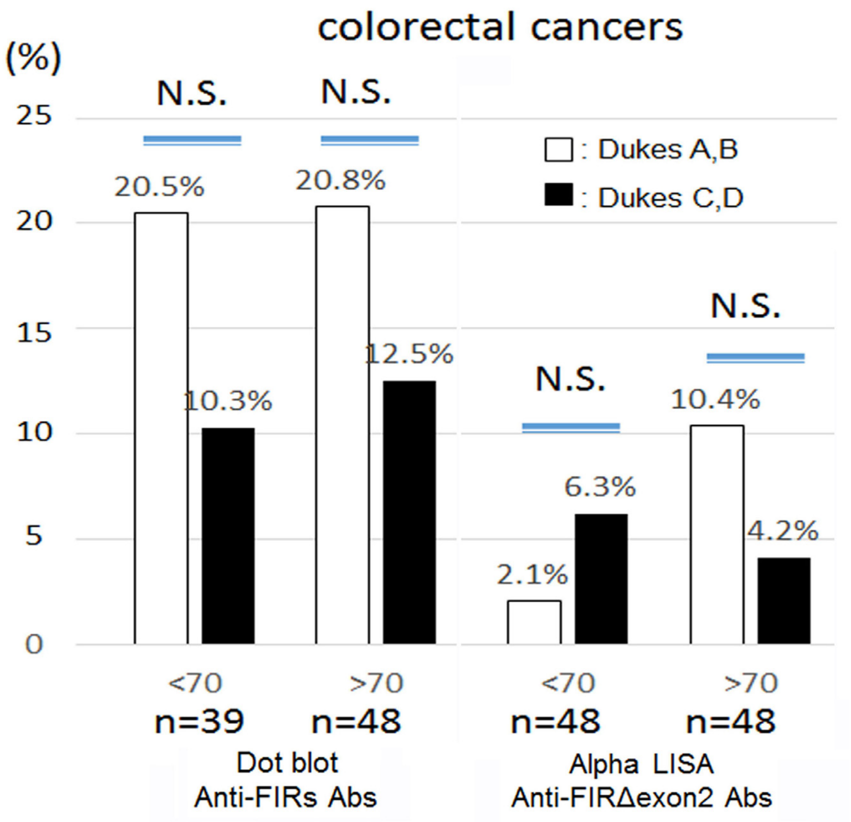

Supplementary Figure S7: Detection rate of anti-FIRs or anti-FIRΔexon2 antibodies in early-stage (Dukes A, B) and advanced (Dukes C, D) colorectal cancers patients in < 70 or 70 < year-old.

Supplementary Table S1: Clinicopathological characteristics

Colon polyps	Age	Sex	Grade	Site	Size (mm)
	56	M	3	Rectum	40
	61	M	3	Ascending	7
	72	F	5	Rectum	20
	60	M	3	Rectum	10 × 15
	69	M	3, 5	Sigmoid	8, 10

Colorectal cancers	Age	Sex	Dukes stages	Sites
	58	F	C	Cecum
	72	M	D	Sigmoid
	68	M	C	Sigmoid
	64	F	C	Decending
	43	F	C	Rectum
	58	F	C	Cecum
	49	M	B	Rectum
	39	F	C	Rectum
	48	F	C	Decending
	50	M	C	Rectum
	55	M	D	Rectum
	72	M	D	Sigmoid
	68	M	C	Sigmoid
	82	M	C	Ascending
	76	F	C	Ascending
	61	M	C	Rectum
